# Crystal structure of 9,9′-{(1*E*,1′*E*)-[1,4-phenyl­enebis(aza­nylyl­idene)]bis­(methanylyl­idene)}bis­(2,3,6,7-tetra­hydro-1*H*,5*H*-pyrido[3,2,1-*ij*]quinolin-8-ol)

**DOI:** 10.1107/S205698901601344X

**Published:** 2016-09-05

**Authors:** Md. Serajul Haque Faizi, Akram Ali, Vadim A. Potaskalov

**Affiliations:** a Department of Chemistry, College of Science, Sultan Qaboos University, PO Box 36 Al-Khod 123, Muscat, Sultanate of , Oman; b Department of Chemistry, Indian Institute of Technology Kanpur, Kanpur, UP 208 016, India; c Department of General and Inorganic Chemistry, National Technical University of Ukraine, Kyiv Polytechnic Institute, 37 Prospect Peremogy, 03056 Kiev, Ukraine

**Keywords:** crystal structure, julolidine, Schiff base, 8-hy­droxy­julolidine-9-carboxaldehyde, *p*-phenyl­enedi­amine, hydrogen bonding, C—H⋯π inter­actions

## Abstract

The whole mol­ecule of the title compound, C_32_H_34_N_2_O_2_, is generated by inversion symmetry; the central benzene ring being situated about the crystallographic inversion center. The aromatic ring of the julolidine moiety is inclined to the central benzene ring by 33.70 (12)°. There are two intra­molecular O—H⋯N hydrogen bonds in the mol­ecule, generating *S*(6) ring motifs. The conformation about the C=N bonds is *E*. The fused non-aromatic rings of the julolidine moiety adopt half-chair conformations. In the crystal, adjacent mol­ecules are linked by pairs of C—H⋯π inter­actions, forming a ladder-like structure propagating along the *a*-axis direction.

## Abstract 2

The whole mol­ecule of the title compound is generated by inversion symmetry; the central benzene ring being situated about a crystallographic inversion center. The aromatic ring of the julolidine moiety is inclined to the central benzene ring by 33.70 (12)°, and the conformation about the C=N bonds is *E*. There are two intra­molecular O—H⋯N hydrogen bonds in the mol­ecule, generating *S*(6) ring motifs.

## Chemical context   

8-Hy­droxy­julolidine-9-carboxaldehyde is a well-known chromophore used in fluorescence chemosensors; chemosensors with the julolidine moiety are usually soluble in aqueous solutions (Narayanaswamy & Govindaraju, 2012 ▸; Maity *et al.*, 2011 ▸; Na *et al.*, 2013 ▸; Noh *et al.*, 2013 ▸). Compounds containing the julolidine group display chromogenic naked-eye detection of copper, zinc, iron, and aluminium ions as well as fluoride ions (Choi *et al.*, 2015 ▸; Wang *et al.*, 2013*a* ▸,*b* ▸; Kim *et al.*, 2015 ▸; Jo *et al.*, 2015 ▸). There are many reports in the literature on 8-hy­droxy­julolidine-9-carboxaldehyde-based Schiff bases and their applications as sensors for metal ions (Park *et al.*, 2014 ▸; Lee *et al.*, 2014 ▸; Kim *et al.*, 2016 ▸). Intra­molecular C—H⋯N hydrogen bonds have been observed in a julolidine-derived structure (Barbero *et al.*, 2012 ▸). Julolidine dyes exhibiting excited-state intra­molecular proton transfer (Nano *et al.*, 2015 ▸) and julolidine ring-containing compounds are also fluorescent probes for the measurement of cell-membrane viscosity. The present work is a part of an ongoing structural study of Schiff bases and their utilization in the synthesis of new organic and polynuclear coordination compounds (Faizi & Sen 2014 ▸; Faizi *et al.*, 2016 ▸). Recently Choi *et al.* (2016 ▸) have reported on a new chemosensor, similar to the title compound, which is a fluorescent chemosensor for the selective detection of Zn^2+^ in aqueous solution. This was synthesized by a condensation reaction of 8-hy­droxy­julolidine-9-carboxaldehyde with 2-(amino­meth­yl)benzene­amine in ethanol at room temperature. We report herein on the synthesis and crystal structure of the title julolidine derivative.
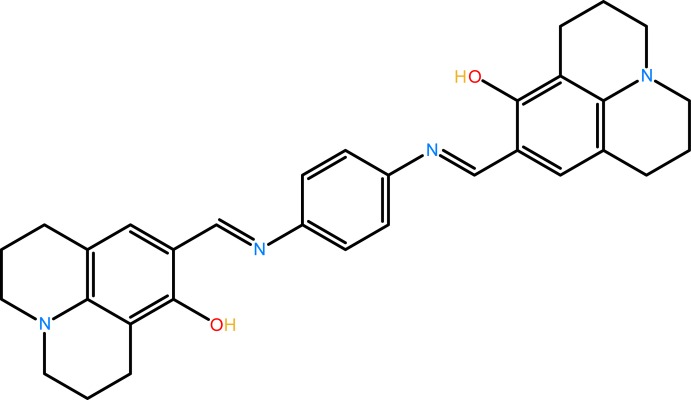


## Structural commentary   

**Figure 1:**
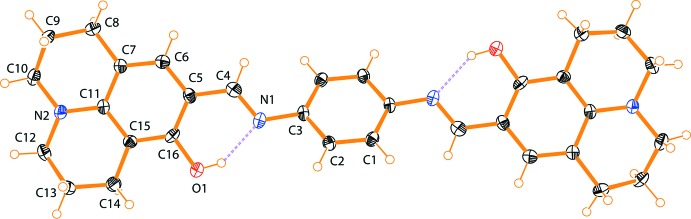
The mol­ecular structure of the title compound, with atom labelling. Displacement ellipsoids are drawn at the 40% probability level. Unlabelled atoms are generated by the symmetry operation −*x*, −*y* + 1, -*z.* The intra­molecular O—H⋯N hydrogen bonds (see Table 1 ▸) are shown as dashed lines.

The mol­ecular structure of the title compound is illustrated in Fig. 1 ▸. The whole mol­ecule of the title compound is generated by crystallographic inversion symmetry. The conformation about the azomethine C4=N1 bond [1.285 (3) Å] is *E*. The C3—N1—C4—C5 torsion angle is 172.9 (2)°. The mol­ecule is non-planar, with the dihedral angle between the central benzene ring and the aromatic ring of the julolidine moiety being 33.70 (12)°. Depending on the tautomers, two types of intra­molecular hydrogen bonds are observed in Schiff bases: O—H⋯N in phenol–imine and N—H⋯O in keto–amine tautomers. The present analysis shows that the title compound exists in the phenol–imine form (Fig. 1 ▸). It exhibits two intra­molecular O1—H1*A*⋯N1 [*d*(N⋯O) 2.579 (3) Å] hydrogen bonds, which generate *S*(6) ring motifs (Fig. 1 ▸ and Table 1 ▸).

## Supra­molecular features   

**Figure 2:**
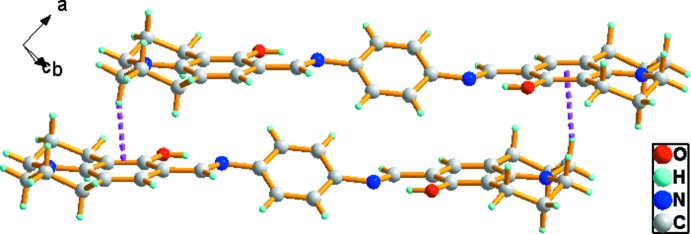
A view of the C—H⋯π inter­actions, shown as dashed lines (see Table 1 ▸), in the crystal of the title compound.

**Figure 3:**
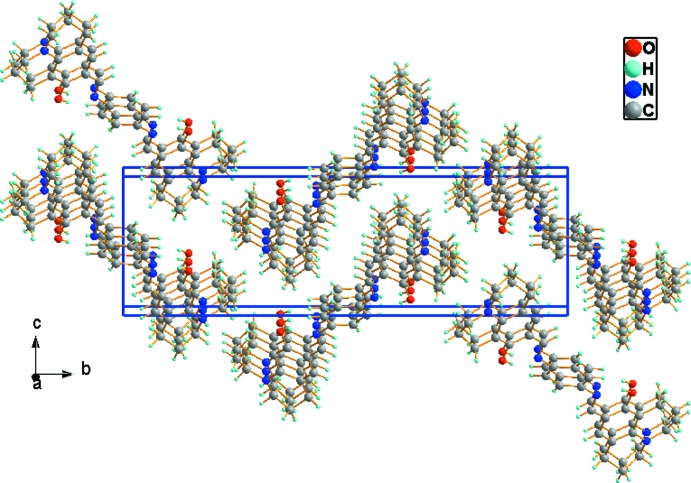
A view along the *a* axis of the crystal packing of the title compound.

In the crystal, adjacent mol­ecules are linked by a pair of C—H⋯π inter­actions (Table 1 ▸ and Fig. 2 ▸), forming a ladder-like structure propagating along the *a*-axis direction (Fig. 3 ▸).

## Database survey   

There are very few examples of similar compounds in the literature and, to the best of our knowledge, the new fluorescent chemosensor for the selective detection of Zn^2+^ in aqueous solution, mentioned in the *Chemical context* section (Choi *et al.*, 2016 ▸) has not been characterized crystallographically. A search of the Cambridge Structural Database (CSD, Version 5.37, update May 2016; Groom *et al.*, 2016 ▸) gave 120 hits for the julolidine moiety. Of these, six have an OH group in position 8, and four also have a C=N group in position 1. Of the latter, one compound, *viz.* 9-{[(4-chlorophen­yl)imino]­meth­yl}-1,1,7,7-tetra­methyl-2,3,6,7-tetra­hydro-1*H*,5*H*-pyrido[3,2,1-*ij*]quinolin-8-ol (CSD refcode: IGALUZ; Kantar *et al.*, 2013 ▸), resembles the title compound and also exists in the phenol–imine form with an intra­molecular O—H⋯N hydrogen bond.

## Synthesis and crystallization   

An ethano­lic solution of 8-hy­droxy­julolidine-9-carboxalde­hyde (100 mg, 0.46 mmol) was added to *p*-phenyl­enedi­amine (25 mg, 0.23 mmol) in absolute ethanol (3 ml). Two drops of HCl were added to the reaction solution and it was stirred for 30 min at room temperature. The resulting yellow precipitate was recovered by filtration, washed several times with small portions of ice-cold EtOH and then with diethyl ether to give 199 mg (85%) of the title compound. Crystals suitable for X-ray diffraction analysis were obtained within three days by slow evaporation of a solution in methanol.

## Refinement   

Crystal data, data collection and structure refinement details are summarized in Table 2 ▸. The OH and C-bound H atoms were included in calculated positions and treated as riding atoms: O—H = 0.82 and C—H = 0.93-0.97 Å, with *U*_iso_(H) = 1.5*U*_eq_(O) and 1.2*U*_eq_(C) for other H atoms.

## Supplementary Material

Crystal structure: contains datablock(s) I. DOI: 10.1107/S205698901601344X/su5322sup1.cif

Structure factors: contains datablock(s) I. DOI: 10.1107/S205698901601344X/su5322Isup2.hkl

Supporting information file. DOI: 10.1107/S205698901601344X/su5322Isup3.cml

CCDC reference: 1500381

Additional supporting information: crystallographic information; 3D view; checkCIF report

## Figures and Tables

**Table 1:** Hydrogen-bond geometry (Å, °) *Cg* is the centroid of the C5–C7/C11/C15/C16 ring.

*D*—H⋯*A*	*D*—H	H⋯*A*	*D*⋯*A*	*D*—H⋯*A*
O1—H1*A*⋯N1	0.82	1.85	2.579 (3)	148
C10—H10*B*⋯*Cg* ^i^	0.97	2.68	3.603 (3)	160

Symmetry code: (i) 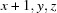.

**Table 2:** Experimental details

Crystal data	
Chemical formula	C_32_H_34_N_4_O_2_
*M*_r_	506.63
Crystal system, space group	Monoclinic, *P*2_1_/*c*
Temperature (K)	100
*a*, *b*, *c* (Å)	5.1776 (3), 27.9346 (17), 8.7893 (6)
β (°)	96.203 (2)
*V* (Å^3^)	1263.79 (14)
*Z*	2
Radiation type	Mo *K*α
μ (mm^−1^)	0.08
Crystal size (mm)	0.20 × 0.15 × 0.12

Data collection	
Diffractometer	Bruker SMART APEX CCD
Absorption correction	Multi-scan (*SADABS*; Bruker, 2003 ▸)
*T*_min_, *T*_max_	0.783, 0.990
No. of measured, independent and observed [*I* > 2σ(*I*)] reflections	15125, 2243, 1469
*R*_int_	0.073
(sin θ/λ)_max_ (Å^−1^)	0.596

Refinement	
*R*[*F* ^2^ > 2σ(*F* ^2^)], *wR*(*F* ^2^), *S*	0.050, 0.127, 1.02
No. of reflections	2243
No. of parameters	173
H-atom treatment	H-atom parameters constrained
Δρ_max_, Δρ_min_ (e Å^−3^)	0.33, −0.22

Computer programs: *SMART* and *SAINT* (Bruker, 2003 ▸), *SIR97* (Altomare *et al.*, 1999 ▸), *DIAMOND* (Brandenberg & Putz, 2006 ▸), *SHELXL97* (Sheldrick, 2008 ▸) and *PLATON* (Spek, 2009 ▸).

## supplementary crystallographic information

### Crystal data

**Table:**

C_32_H_34_N_4_O_2_	*F*(000) = 540
*M**_r_* = 506.63	*D*_x_ = 1.331 Mg m^−^^3^
Monoclinic, *P*2_1_/*c*	Mo *K*α radiation, λ = 0.71073 Å
Hall symbol: -P 2ybc	Cell parameters from 3371 reflections
*a* = 5.1776 (3) Å	θ = 2.4–26.5°
*b* = 27.9346 (17) Å	µ = 0.08 mm^−^^1^
*c* = 8.7893 (6) Å	*T* = 100 K
β = 96.203 (2)°	Block, yellow
*V* = 1263.79 (14) Å^3^	0.20 × 0.15 × 0.12 mm
*Z* = 2	

### Data collection

**Table:**

Bruker SMART APEX CCD diffractometer	2243 independent reflections
Radiation source: fine-focus sealed tube	1469 reflections with *I* > 2σ(*I*)
Graphite monochromator	*R*_int_ = 0.073
/w–scans	θ_max_ = 25.0°, θ_min_ = 2.8°
Absorption correction: multi-scan (SADABS; Bruker, 2003)	*h* = −6→6
*T*_min_ = 0.783, *T*_max_ = 0.990	*k* = −33→33
15125 measured reflections	*l* = −10→10

### Refinement

**Table:**

Refinement on *F*^2^	Primary atom site location: structure-invariant direct methods
Least-squares matrix: full	Secondary atom site location: difference Fourier map
*R*[*F*^2^ > 2σ(*F*^2^)] = 0.050	Hydrogen site location: inferred from neighbouring sites
*wR*(*F*^2^) = 0.127	H-atom parameters constrained
*S* = 1.02	*w* = 1/[σ^2^(*F*_o_^2^) + (0.0483*P*)^2^ + 0.7868*P*] where *P* = (*F*_o_^2^ + 2*F*_c_^2^)/3
2243 reflections	(Δ/σ)_max_ < 0.001
173 parameters	Δρ_max_ = 0.33 e Å^−^^3^
0 restraints	Δρ_min_ = −0.22 e Å^−^^3^

### Special details

**Table:**

Geometry. All esds (except the esd in the dihedral angle between two l.s. planes) are estimated using the full covariance matrix. The cell esds are taken into account individually in the estimation of esds in distances, angles and torsion angles; correlations between esds in cell parameters are only used when they are defined by crystal symmetry. An approximate (isotropic) treatment of cell esds is used for estimating esds involving l.s. planes.
Refinement. Refinement of F^2^ against ALL reflections. The weighted R-factor wR and goodness of fit S are based on F^2^, conventional R-factors R are based on F, with F set to zero for negative F^2^. The threshold expression of F^2^ > 2sigma(F^2^) is used only for calculating R-factors(gt) etc. and is not relevant to the choice of reflections for refinement. R-factors based on F^2^ are statistically about twice as large as those based on F, and R- factors based on ALL data will be even larger.

### Fractional atomic coordinates and isotropic or equivalent isotropic displacement parameters (Å^2^)

**Table:**

	*x*	*y*	*z*	*U*_iso_*/*U*_eq_	
O1	0.5852 (3)	0.64668 (6)	0.15270 (19)	0.0327 (5)	
H1A	0.4785	0.6259	0.1246	0.049*	
N2	1.2746 (4)	0.67853 (7)	0.5427 (2)	0.0250 (5)	
N1	0.3470 (4)	0.56564 (7)	0.1548 (2)	0.0259 (5)	
C3	0.1732 (4)	0.53146 (8)	0.0816 (3)	0.0216 (6)	
C11	1.0954 (4)	0.64728 (8)	0.4704 (3)	0.0208 (6)	
C7	1.0677 (4)	0.60049 (8)	0.5312 (3)	0.0212 (6)	
C1	−0.2269 (4)	0.51736 (9)	−0.0764 (3)	0.0249 (6)	
H1	−0.3807	0.5292	−0.1271	0.030*	
C15	0.9341 (4)	0.66163 (8)	0.3388 (3)	0.0229 (6)	
C16	0.7394 (5)	0.63101 (9)	0.2775 (3)	0.0250 (6)	
C6	0.8730 (4)	0.57163 (9)	0.4650 (3)	0.0245 (6)	
H6	0.8546	0.5412	0.5055	0.029*	
C2	−0.0546 (4)	0.54825 (9)	0.0030 (3)	0.0240 (6)	
H2	−0.0918	0.5808	0.0039	0.029*	
C5	0.7015 (5)	0.58567 (8)	0.3400 (3)	0.0241 (6)	
C4	0.5029 (5)	0.55395 (9)	0.2728 (3)	0.0277 (6)	
H4	0.4865	0.5239	0.3161	0.033*	
C8	1.2531 (5)	0.58325 (9)	0.6635 (3)	0.0277 (6)	
H8A	1.3963	0.5663	0.6250	0.033*	
H8B	1.1643	0.5611	0.7251	0.033*	
C12	1.3361 (5)	0.72291 (9)	0.4669 (3)	0.0305 (6)	
H12A	1.4648	0.7165	0.3971	0.037*	
H12B	1.4101	0.7456	0.5429	0.037*	
C10	1.4682 (5)	0.66195 (9)	0.6634 (3)	0.0305 (6)	
H10A	1.5310	0.6889	0.7264	0.037*	
H10B	1.6144	0.6484	0.6181	0.037*	
C14	0.9740 (5)	0.70887 (9)	0.2652 (3)	0.0307 (6)	
H14A	0.8078	0.7212	0.2203	0.037*	
H14B	1.0841	0.7046	0.1837	0.037*	
C13	1.0976 (5)	0.74444 (9)	0.3793 (3)	0.0313 (6)	
H13A	1.1453	0.7730	0.3264	0.038*	
H13B	0.9744	0.7535	0.4499	0.038*	
C9	1.3577 (5)	0.62498 (9)	0.7617 (3)	0.0309 (6)	
H9A	1.2190	0.6390	0.8130	0.037*	
H9B	1.4917	0.6138	0.8393	0.037*	

### Atomic displacement parameters (Å^2^)

**Table:**

	*U*^11^	*U*^22^	*U*^33^	*U*^12^	*U*^13^	*U*^23^
O1	0.0348 (11)	0.0320 (11)	0.0288 (10)	−0.0051 (8)	−0.0072 (9)	0.0013 (8)
N2	0.0219 (11)	0.0267 (12)	0.0257 (12)	−0.0030 (10)	−0.0006 (9)	−0.0002 (9)
N1	0.0213 (11)	0.0324 (13)	0.0233 (11)	−0.0016 (10)	−0.0003 (10)	−0.0044 (10)
C3	0.0198 (12)	0.0261 (13)	0.0198 (13)	−0.0057 (11)	0.0060 (11)	−0.0059 (11)
C11	0.0174 (12)	0.0239 (14)	0.0219 (13)	−0.0019 (10)	0.0057 (11)	−0.0050 (10)
C7	0.0213 (13)	0.0239 (14)	0.0192 (13)	0.0009 (11)	0.0061 (11)	−0.0041 (11)
C1	0.0187 (13)	0.0316 (15)	0.0241 (14)	0.0004 (11)	0.0016 (11)	−0.0006 (11)
C15	0.0248 (13)	0.0244 (13)	0.0202 (13)	−0.0008 (11)	0.0049 (11)	0.0019 (11)
C16	0.0229 (13)	0.0350 (15)	0.0165 (12)	0.0061 (12)	−0.0010 (11)	−0.0004 (11)
C6	0.0273 (14)	0.0252 (14)	0.0217 (13)	0.0020 (11)	0.0065 (11)	−0.0004 (11)
C2	0.0239 (13)	0.0233 (14)	0.0251 (14)	−0.0017 (11)	0.0042 (11)	−0.0038 (11)
C5	0.0263 (14)	0.0236 (14)	0.0234 (14)	−0.0039 (11)	0.0070 (12)	−0.0052 (11)
C4	0.0312 (14)	0.0263 (14)	0.0270 (14)	0.0006 (12)	0.0098 (12)	−0.0024 (12)
C8	0.0297 (15)	0.0305 (15)	0.0229 (14)	0.0056 (12)	0.0030 (12)	0.0031 (11)
C12	0.0275 (14)	0.0250 (14)	0.0394 (16)	−0.0064 (12)	0.0048 (12)	−0.0054 (12)
C10	0.0253 (13)	0.0350 (15)	0.0296 (15)	0.0023 (12)	−0.0043 (12)	−0.0082 (12)
C14	0.0289 (14)	0.0323 (15)	0.0305 (15)	−0.0034 (12)	0.0015 (12)	0.0033 (12)
C13	0.0356 (15)	0.0255 (14)	0.0329 (15)	−0.0034 (12)	0.0047 (13)	0.0045 (12)
C9	0.0292 (14)	0.0384 (16)	0.0233 (14)	0.0096 (13)	−0.0056 (11)	−0.0031 (12)

### Geometric parameters (Å, º)

**Table:**

O1—C16	1.358 (3)	C6—H6	0.9300
O1—H1A	0.8200	C2—H2	0.9300
N2—C11	1.378 (3)	C5—C4	1.435 (3)
N2—C10	1.454 (3)	C4—H4	0.9300
N2—C12	1.459 (3)	C8—C9	1.515 (3)
N1—C4	1.285 (3)	C8—H8A	0.9700
N1—C3	1.418 (3)	C8—H8B	0.9700
C3—C2	1.383 (3)	C12—C13	1.508 (3)
C3—C1^i^	1.394 (3)	C12—H12A	0.9700
C11—C15	1.410 (3)	C12—H12B	0.9700
C11—C7	1.425 (3)	C10—C9	1.499 (4)
C7—C6	1.370 (3)	C10—H10A	0.9700
C7—C8	1.505 (3)	C10—H10B	0.9700
C1—C2	1.376 (3)	C14—C13	1.505 (3)
C1—C3^i^	1.394 (3)	C14—H14A	0.9700
C1—H1	0.9300	C14—H14B	0.9700
C15—C16	1.386 (3)	C13—H13A	0.9700
C15—C14	1.494 (3)	C13—H13B	0.9700
C16—C5	1.403 (3)	C9—H9A	0.9700
C6—C5	1.392 (3)	C9—H9B	0.9700
			
C16—O1—H1A	109.5	C7—C8—H8A	109.5
C11—N2—C10	120.75 (19)	C9—C8—H8A	109.5
C11—N2—C12	119.8 (2)	C7—C8—H8B	109.5
C10—N2—C12	115.88 (19)	C9—C8—H8B	109.5
C4—N1—C3	120.5 (2)	H8A—C8—H8B	108.1
C2—C3—C1^i^	118.6 (2)	N2—C12—C13	111.4 (2)
C2—C3—N1	117.6 (2)	N2—C12—H12A	109.3
C1^i^—C3—N1	123.7 (2)	C13—C12—H12A	109.3
N2—C11—C15	120.5 (2)	N2—C12—H12B	109.3
N2—C11—C7	119.9 (2)	C13—C12—H12B	109.3
C15—C11—C7	119.6 (2)	H12A—C12—H12B	108.0
C6—C7—C11	118.7 (2)	N2—C10—C9	111.4 (2)
C6—C7—C8	121.2 (2)	N2—C10—H10A	109.4
C11—C7—C8	120.1 (2)	C9—C10—H10A	109.4
C2—C1—C3^i^	120.6 (2)	N2—C10—H10B	109.4
C2—C1—H1	119.7	C9—C10—H10B	109.4
C3^i^—C1—H1	119.7	H10A—C10—H10B	108.0
C16—C15—C11	119.0 (2)	C15—C14—C13	111.3 (2)
C16—C15—C14	120.4 (2)	C15—C14—H14A	109.4
C11—C15—C14	120.6 (2)	C13—C14—H14A	109.4
O1—C16—C15	117.0 (2)	C15—C14—H14B	109.4
O1—C16—C5	120.8 (2)	C13—C14—H14B	109.4
C15—C16—C5	122.1 (2)	H14A—C14—H14B	108.0
C7—C6—C5	123.1 (2)	C12—C13—C14	110.0 (2)
C7—C6—H6	118.4	C12—C13—H13A	109.7
C5—C6—H6	118.4	C14—C13—H13A	109.7
C1—C2—C3	120.8 (2)	C12—C13—H13B	109.7
C1—C2—H2	119.6	C14—C13—H13B	109.7
C3—C2—H2	119.6	H13A—C13—H13B	108.2
C6—C5—C16	117.3 (2)	C10—C9—C8	109.7 (2)
C6—C5—C4	121.2 (2)	C10—C9—H9A	109.7
C16—C5—C4	121.4 (2)	C8—C9—H9A	109.7
N1—C4—C5	122.3 (2)	C10—C9—H9B	109.7
N1—C4—H4	118.8	C8—C9—H9B	109.7
C5—C4—H4	118.8	H9A—C9—H9B	108.2
C7—C8—C9	110.7 (2)		

Symmetry code: (i) −*x*, −*y*+1, −*z*.

### Hydrogen-bond geometry (Å, º)

Cg is the centroid of the C5–C7/C11/C15/C16 ring.

**Table:**

*D*—H···*A*	*D*—H	H···*A*	*D*···*A*	*D*—H···*A*
O1—H1*A*···N1	0.82	1.85	2.579 (3)	148
C10—H10*B*···*Cg*^ii^	0.97	2.68	3.603 (3)	160

Symmetry code: (ii) *x*+1, *y*, *z*.
